# An In-Vitro Analysis of Peri-Implant Mucosal Seal Following Photofunctionalization of Zirconia Abutment Materials

**DOI:** 10.3390/biomedicines9010078

**Published:** 2021-01-15

**Authors:** Masfueh Razali, Wei Cheong Ngeow, Ros Anita Omar, Wen Lin Chai

**Affiliations:** 1 Department of Restorative Dentistry, Faculty of Dentistry, Universiti Kebangsaan Malaysia, Kuala Lumpur 50300, Malaysia; 2 Department of Restorative Dentistry, Faculty of Dentistry, University of Malaya, Kuala Lumpur 50603, Malaysia; anitaomar@um.edu.my; 3 Department of Oral and Maxillofacial Clinical Sciences, Faculty of Dentistry, University of Malaya, Kuala Lumpur 50603, Malaysia; ngeowy@um.edu.my

**Keywords:** biological seal, ultraviolet, zirconia abutments, photofunctionalization, implant-soft tissue interface, organotypic model, three-dimensional peri-implant mucosal model

## Abstract

The presence of epithelial and connective tissue attachment at the peri-implant–soft tissue region has been demonstrated to provide a biological barrier of the alveolar bone from the oral environment. This barrier can be improved via surface modification of implant abutment materials. The effect of photofunctionalization on creating a bioactive surface for the enhancement of the epithelial and connective tissue attachment of zirconia implant abutment’s peri-implant mucosal interface using organotypic model has not been investigated. Therefore, this study aimed to evaluate the soft tissue seal around peri-implant mucosa and to understand the effect of photofunctionalization on the abutment materials. Three types of abutment materials were used in this study; yttria-stabilized zirconia (YSZ), alumina-toughened zirconia, and grade 2 commercially pure titanium (CPTi) which were divided into nontreated (N-Tx) and photofunctionalized group (UV-Tx). The three-dimensional peri-implant mucosal model was constructed using primary human gingival keratinocytes and fibroblasts co-cultured on the acellular dermal membrane. The biological seal was determined through the concentration of tritiated water permeating the material–soft tissue interface. The biological seal formed by the soft tissue in the N-Tx group was significantly reduced compared to the UV-treated group (*p* < 0.001), with YSZ exhibiting the lowest permeability among all materials. Photofunctionalization of implant abutment materials improved the biological seal of the surrounding soft tissue peri-implant interface.

## 1. Introduction

The standard material used for manufacturing dental implants is grade 2 and 4 commercially pure titanium (CPTi). This material has displayed a high success rate and has shown to be biocompatible and resistant to corrosion by forming an inert titanium oxide surface layer. The estimated survival rate for 10 years of an implant is 96.4% (95% confidence interval 95.2–97.5%) [1], and the clinical long-term success rate of titanium-based dental implants over a follow-up period of 36 years is estimated at 87.8% [2]. Nevertheless, several studies have proven that titanium can negatively influence cell metabolism [3] and cause some adverse effects, metal allergy (such as delayed hypersensitive reaction) [4,5] and is responsible for oral biofilm dysbiosis [6] as well as the induction of oxidative stress [7] when trace metal ions are released by the titanium in the body. The use of titanium implants or abutments can also be a disadvantage—from an aesthetics perspective, they can shine through thin gingiva, particularly when smiling. As the dental material industry evolves, the white-opaque nature of zirconia provides good aesthetics [8,9], high tensile strength [10] and biocompatibility [11] as dental implants or implant abutments. Over the past decade, zirconia has been used as implant abutments because of the biomechanical and biological qualities of the material and its aesthetic advantage; thus, it is suitable in thin gingiva or in cases of soft-tissue recessions [12,13].

The ability of ultraviolet (UV) radiation to counter the aging of a titanium implant [14,15] and remove nonbiological contaminants [16] on the implant surface has previously been reported [15]. This mode of surface modification is known as photofunctionalization, a surface conditioning of dental implants via UV irradiation that can easily be carried out at the chairside. UV irradiation of the implant surface has been reported to enhance the wettability of the implant surface and promote fast bone healing [17,18]. The effect of photofunctionalization on titanium in improving osseointegration has been documented in animals [18,19,20] and human studies [21,22]. Nevertheless, the effect on the peri-implant tissue is not well described in the literature.

Similar to titanium, zirconia is a semiconductor with a wide bandgap and excellent flat band potentials (i.e., better reduction potential from the valance band to the conduction band) [23]. In the presence of UV light, zirconia is also able to undergo photocatalytic reaction since it contains extra electronic levels at intermediate energy in the bandgap, capable of allowing the transition of electrons from the valence band to the conduction band with a double excitation. Unlike titanium, zirconia has a bandgap energy wider than that of titanium, hence requires larger photon energy (about 5.0 eV to energize the electron) [24]. Following UV irradiation, the surface oxygen vacancies at the bridging sites of photocatalytic reaction of zirconia creates conversion of relevant Zr^4+^ sites to Zr^3+^ sites, resulting in increased in wettability [23,25].

Moreover, the positively charged zirconia surface directly interacts with the negatively charged biological cells. Similar to titanium, irradiation of zirconia with UV light results in the excitation of an electron from the valence band to the conduction band of zirconia, thereby producing negative-electron (e^−^) and positive-hole (h^+^) pairs. The positive hole on the superficial layer of zirconia increases the surface free energy to become more electropositive. The divalent cations following UV-treated zirconia surfaces act as direct attractants for cells, and the positively charged zirconia surface can attach directly to negatively charged proteins and cells, making it more bioactive. In previous studies, UV irradiation has been shown to create an amphiphilic and hydrophilic surface on zirconia and remove hydrocarbon contaminations or bacteria found attached to the implants [25,26,27]. Henceforward, photofunctionalization is important for the bioactivity of zirconia dental implants. However, in general, limited research is available in understanding the effect of surface modifications toward optimizing zirconia abutments for better soft tissue contact. This limitation applies particularly to the improvement of peri-implant mucosal biological seal in response to the photofunctionalization of the implant-abutment material, thereby highlighting the importance and necessity of conducting more research in this field.

Based on previous reports, the commonly used in vitro techniques for the evaluation of the implant–soft tissue interaction are primarily based on monolayer culture of keratinocytes [28] or fibroblasts [29] on zirconia surfaces. Therefore, this study aimed to analyze the biological seal formed by connective tissue and epithelial attachment using a three-dimensional peri-implant mucosal model following photofunctionalization of selected dental implant abutment materials.

## 2. Materials and Methods

### 2.1. Preparation of Specimens

Three types of abutment materials were used in this study: (1) fully yttria-stabilized zirconia (YSZ), obtained from Safe Implant^®^ (Best Tools Manufacturing Co., Ltd., Bangkok, Thailand); and (2) alumina-toughened zirconia (ATZ) prepared from Zeramex^®^ P6 (Dentalpoint AG, Spreitenbach, Switzerland) and (3) CPTi (Edgetech Industries LLC, Miramar, FL, USA) (grade 2) as control. All the specimens were supplied with a specific dimension to meet the technical requirements of the experiments, disks with a dimension of 5 mm diameter and 3 mm thickness, while the surface roughness was categorized within the smooth surface. The specimens were used as received from the suppliers. In this study, a total of 18 specimens were used, where six specimens represent each material type.

These specimens were evaluated for surface roughness prior to sterilization processes. The surface roughness of all specimens was evaluated and standardized to be within the range of the smooth surface values (S_a_ < 0.5 µm/500 nm) [30]. Surface roughness denoted as S_a_ was evaluated using an atomic force microscope (AFM; Ambios Q-Scope, Ambios Technology Inc., Santa Cruz, CA, USA) in contact mode. In brief, three random points of disks’ side were measured and averaged. The expected values of S_a_ of all specimens selected in the experiments were within the smooth surface category.

All samples were washed with deionized water for 15 min in an ultrasonic bath and sterilized using an ultrasonic rinse with ascending concentration of ethanol (50%, 70%, and 100%) for 5 min. The samples were air-dried and kept in a sterilized glass container before any experiment was carried out.

**Figure 1:**
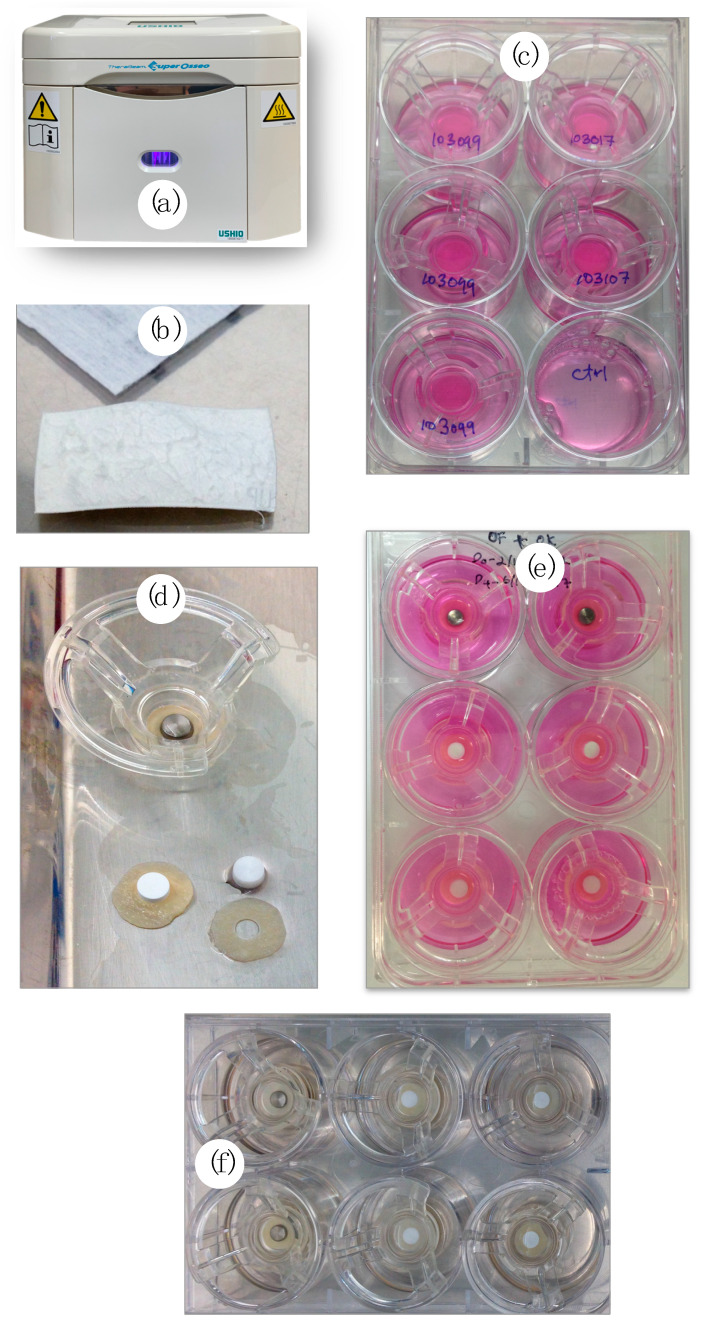
Steps in preparing the three-dimensional peri-implant mucosal model (**a**) the UV generating device; (**b**) membrane; (**c**) the membrane was cut into a round shape, fit in the insert with cells inoculated; (**d**) a punch hole was created in the middle of membrane and specimen inserted; (**e**) the tissue and specimens were incubated for up to ten days; (**f**) the tissues were washed with Dulbecco’s phosphate buffered saline before a permeability test was carried out.

These specimens were further equally divided into the nontreated (N-Tx) and photofunctionalized (UV-Tx) groups for the experiment to be carried out. UV-light treatment was performed using a UV-light device (Therabeam^®^ SuperOsseo, Ushio, Tokyo, Japan) (Figure 1a) for 12 min. The device generated a mixture of spectra; intensity was 0.05 mW/cm^2^ (λ = 360 nm) and 2 mW/cm^2^ (λ = 250 nm). These parameters were standardized and set by manufacturers for optimum titanium UV exposure.

### 2.2. Cell Extraction

Human primary gingival cells were used to construct the three-dimensional peri-implant mucosal model (3D-PIMM), an organotypic model that mimics the actual peri-implant mucosa. The primary cells, that is, human gingival keratinocytes (HGK) and human gingival fibroblasts (HGF), were obtained from biopsies of healthy gingival tissues of patients who underwent surgical crown lengthening procedures. Ethical approval was obtained from the Medical Ethics Committee Faculty of Dentistry, University of Malaya (DF DP1406/0061(L)), for using human waste oral tissues for the reconstruction of the 3D-PIMM. Patients consented for the tissues to be obtained in this study.

Primary cells were extracted from the gingival tissues using an explant technique. The epithelial and connective tissue were separated using protease enzyme. The separated tissues were minced using a scalpel blade in separate petri dishes and transferred to culture flasks. The growth media in the flasks were changed every 2–3 days until the cell reached its confluence. When using the cells, they were washed with Dulbecco’s phosphate-buffered saline (DPBS) (Thermo Fisher Scientific, Inc., Waltham, MA, USA), dissociated and resuspended for further use in the 3D-PIMM.

A total of 3.0 × 10^6^ cells (for both primary HGK and HGF) were needed in each experiment to construct six 3D-PIMM. Cell extraction, which was carried out in this experiment, yielded approximately 1.5 × 10^6^ cells from one donor; therefore, two donors were essentially required per experiment. The cells from both donors were mixed prior to cell count to avoid variation in cell growth and proliferation because of differences in donors.

### 2.3. Three-Dimensional Peri-Implant Mucosal Model Development

A 3D-PIMM was developed for implant–soft tissue interface investigation based on the modification from a previous model [31]. The procedures carried out are depicted in Figure 1. An acellular human cadaveric dermis (Alloderm^®^ RTM, LifeCell Corporation, Branchburg, NJ, USA) (Figure 1b) was cut into a 12-mm diameter circle to fit into the ring insert of similar diameter (Snapwell^™^ Insert, Corning Life Sciences, Corning, NY, USA). Following rehydration with phosphate buffer saline (PBS) (Thermo Fisher Scientific, Inc., Waltham, MA, USA) and Green’s medium, it was placed into 12-mm-diameter inserts with the basement membrane side up. The above-mentioned primary HGK and HGF cell suspension was mixed and co-cultured onto the basement membrane at a density of 500,000 each cell (Figure 1c). These models were incubated at 37 °C in an atmosphere of 5% CO_2_. The media were changed every 2 days both in the insert and well with Green’s media. These models were incubated at 37 °C in an atmosphere of 5% CO_2_. The media were changed every two days both in the insert and well with Green’s media.

On the fourth day, a 4-mm-diameter hole was prepared using a sterile disposable tissue biopsy punch precisely in the middle of the membrane (Kai Industries Co., Ltd., Gifu, Japan). A specimen disk (5 mm diameter × 3.0 mm height) was then placed into the punched hole (Figure 1d). In each six-well plate, three groups of material (treated and nontreated with UV light) were placed (Figure 1e). The tissues were lifted at the air–liquid interface (ALI) to promote epithelial stratification after 10 days of culture. The punch tissue (4 mm) obtained from the hole preparation was grown in a 24-well plate parallel to the 3D-PIMM, with a media change every 2 days. Cell growth was constantly monitored.

### 2.4. Permeability Test

**Figure 2:**
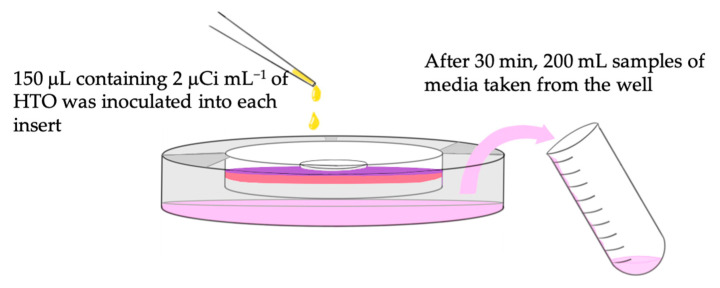
Schematic illustration of the procedure collecting the tritiated water (HTO).

Tritiated water or the radioactive form of water (HTO) (^3^H, Packard, PerkinElmer Inc., Hopkinton, MA, USA), was used as a tracing agent to evaluate the permeability of the model. Permeability test was performed at room temperature on day 14 of the tissue models. The media in the insert were removed, and the inserts were washed and transferred to a new six-well plate containing 3 mL of new complete DMEM in each well (Figure 1f). An aliquot of 150 μL containing 2 μCi mL^−1^ of HTO was inoculated into each insert. After 30 min, a 200 mL of sample media in the well was obtained and labeled. The samples were mixed with 3.0 mL of scintillation cocktail (Ultima Gold™ XR, PerkinElmer Inc., Santa Clara, CA, USA), and the radioactivity of the samples (the measurement of tritiated water in media) was counted for 15 min using a liquid scintillation counter (Liquid Scintillation Analyzer Packard TRI-CARB Model 2700, Packard Instrument Company Inc., Meriden, CT, USA). The schematic description of the procedure is illustrated in Figure 2. The raw data obtained from the scintillation counter were the amount of HTO radioactivity that has penetrated through the tissue and material interface.

The concentration of radioactivity measured was expressed in percentage using the following formula:

Amount X (%) = Q_x_/Q_o_ × 100% (1)

where Q_x_ is the radioactivity of HTO penetrating through the model (counts per minute, cpm), and Q_o_ is the amount of radioactivity in 150 μL containing 2 μCi mL^−1^ of HTO in a 200 mL media, without passing through any membrane.

From the amount of radioactivity that penetrated through, the steady state of liquid influx through the interface at a given time can also be measured. Based on Fick’s first law of diffusion [32] the steady-state flux (J_ss_) was calculated using the following formula:

J_ss_ = Q/A*t* (2)

where Q is the radioactivity of HTO (radioactivity count) penetrating through the model–material interface; A is the area of exposed tissue (cm^2^), and *t* = time (min) given for the tritiated water to pass through the membrane and interface. The area of exposed tissue A calculated using the area of the membrane, which was the area covered by the material, is derived as follows:

A = π(R_m_ − R_d_)^2^ (3)

where R_m_ is the radius of the membrane, and R_d_ is the radius of the disk specimens. The unit was given as J_ss_ = count/cm^2^/min.

### 2.5. Histological Preparation

The remaining soft tissues were fixed with 4% phosphate-buffered formalin solution (pH 7.2) for 3 days, processed for histological sections via dehydration in a graded series of ethanol concentrations (70–100%), and finally embedded in a paraffin block. The blocks were thinly sectioned and stained with hematoxylin and eosin staining (H&E staining). The punch tissues (4 mm) obtained from the hole preparation described earlier were also fixed in 4% formalin and further processed for H&E staining.

### 2.6. Statistical Analysis

All experiments were conducted in triplicate. The quantitative data were expressed as mean ± standard deviation. The collected data were analyzed with IBM SPPS (Statistical Package of Social Science) for MacOS Catalina, version 26.0. The Shapiro–Wilk normality test was used to determine normally distributed data with *p*-values > 0.05 and Levene’s test was used to test the assumption of homogeneity of variance. A one-way analysis of variance (ANOVA) was used to compare the surface topography among different materials. A two-way ANOVA with a post-hoc Tukey was used to analyze both of the permeability potential and steady-state flux of different materials against UV treatment. The hypothesis of no difference was accepted when *p*-values > 0.05.

## 3. Results

### 3.1. Composition and Surface Roughness

**Figure 3:**
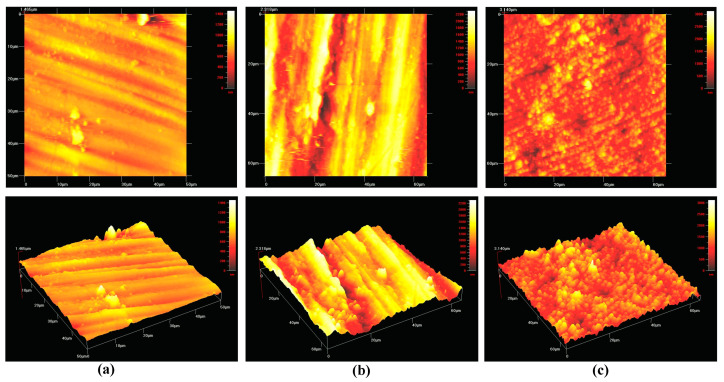
Surface topography of each material: (**a**) commercially pure titanium; (**b**) yttria-stablized zirconia; and (**c**) aluminium-toughened zirconia. Scale bar (**a**) 0–1400 nm; (**b**) 0–2200 nm; and (**c**) 0–3000 nm.

The surface roughness of all specimens is tabulated in Table 1. All material surfaces were categorized within the range of the smooth surface values (S_a_ < 0.5 μm) with no statistically significant difference (*p*-value > 0.05) being observed among the different types of materials. Figure 3 shows the topographic surface profile of each material used in this study, as scanned by atomic force microscopy (AFM).

### 3.2. Cell Extraction and 3D Peri-Implant Mucosal Model

**Figure 4:**
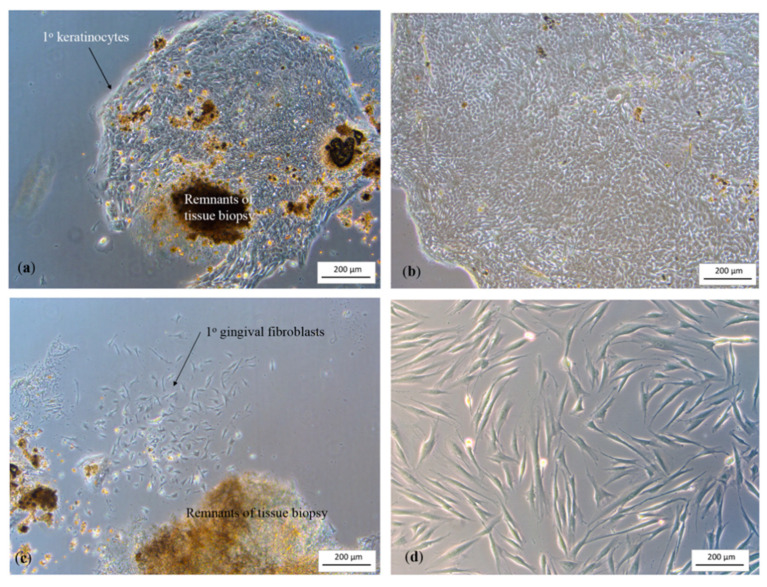
(**a**) The primary human gingival keratinocytes migrated out from the tissue and formed a colony as observed on day 7; (**b**) the HGK reached 80% confluence; (**c**) the primary human gingival fibroblasts presented at the adjacent of the tissue biopsy after 4 days of culture; (**d**) the primary HGF were proliferated and evenly distributed within the flask.

Primary cell growth was constantly monitored. Figure 4a–d show the images of primary HGK and HGF observed a few days following cell extraction. The black spots in the images were the remnants of tissue biopsies, which eventually detached from the flasks during media change.

### 3.3. Permeability Test

Table 2 shows the mean percentages of permeability of different materials following UV light surface treatment. Notably, untreated CPTi allowed more HTO to penetrate the interface than other materials, whereas the treated groups have reduced mean percentage of HTO. A statistically significant difference was found between N-Tx and UV-Tx (*p* = 0.002) for all types of materials. However, when comparison was made amongst materials, there was a statistically significant difference between YSZ and CPTi (*p* < 0.001) and between YSZ and ATZ (*p* = 0.017), but no significant difference was observed between CPTi and ATZ (*p* = 0.061).

**Figure 5:**
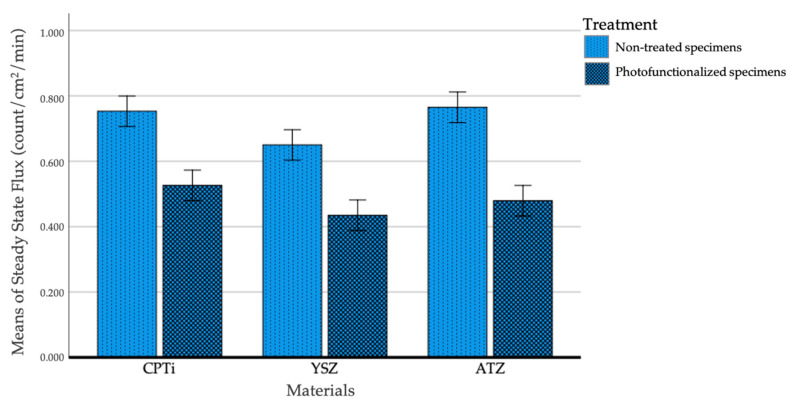
Mean steady-state flux (Jss) of penetrated HTO through the soft tissue–material interface. The error bars represent a standard error at a 95% confidence interval.

Figure 5 shows the steady-state flux of HTO through the tissue–material interface. The flux across the interface for all materials was statistically and significantly low following UV treatment (*p* < 0.001) without interaction among materials. When multiple comparisons test was performed, a significant difference was observed between YSZ and CPTi (*p* = 0.001) and between YSZ and ATZ (*p* = 0.004), but no significant difference was found between CPTI and ATZ (*p* = 0.727). Based on this result, YSZ exhibited the lowest permeability, as demonstrated by the low steady-state flux at a given time.

### 3.4. Histological Analyses

**Figure 6:**
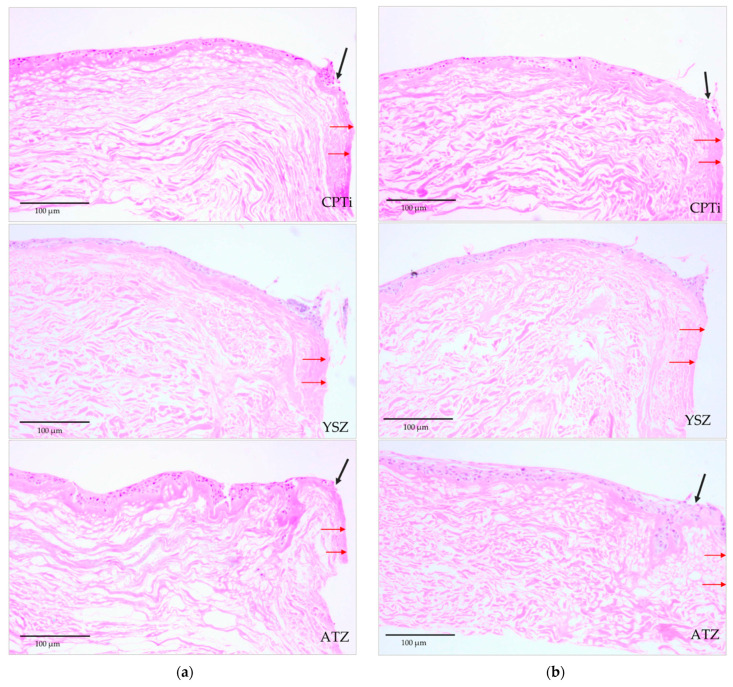
Tissue model following detachment of the disk (10× magnification); (**a**) UV-treated specimen–soft tissue interface and (**b**) nontreated specimen–soft tissue interface showing a few layers of epithelial cell formed and distortion of cell layers at the soft tissue–disk interface (shown in black arrows) for each type of materials. The soft tissue–specimen interface is indicated by red arrows.

**Figure 7:**
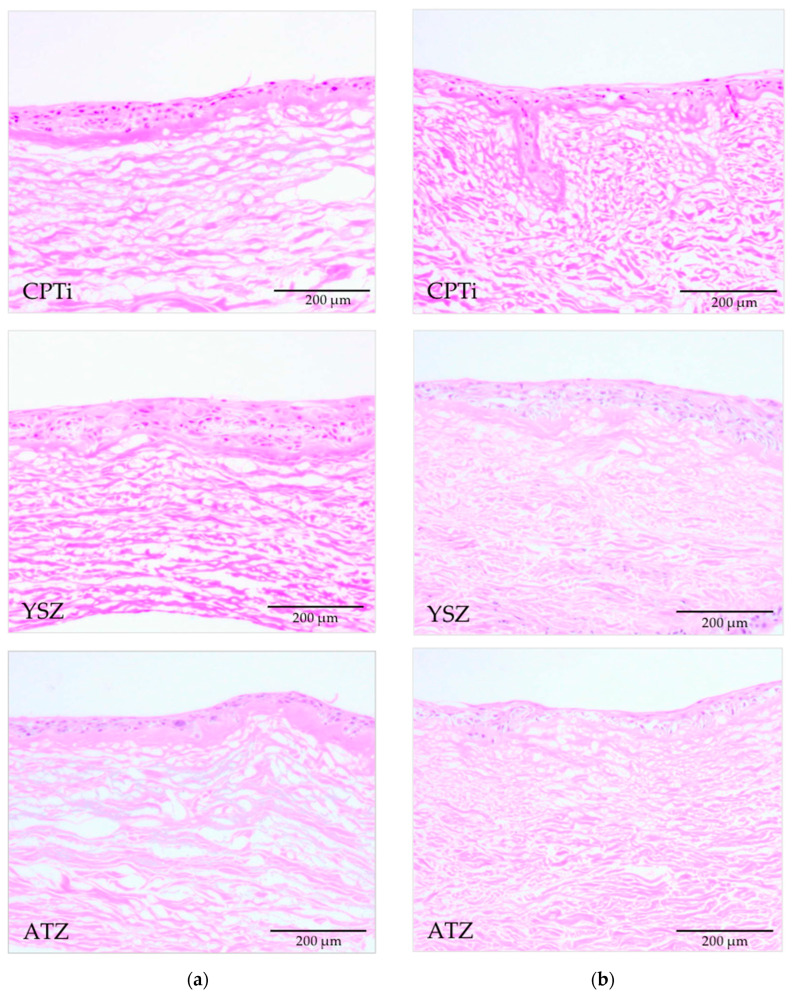
Histological section of the corresponding punched tissue of the models (**a**) from the UV-treated and (**b**) from the nontreated groups (20× magnification).

Histological sections of the 3D-PIMM were examined under a light microscope. They consisted of well-formed, stratified squamous epithelium with four to six layers of epithelial cells, as shown in Figure 6. The black arrows indicate the distortion of epithelial layers during the pull-out procedure, and red arrows indicate the specimen–soft tissue interface. Figure 7 shows the histological sections of the punched tissues, which were grown parallel to the 3D-PIMM. The epithelial stratification of varying thickness was noted in all histological sections. The presence of epithelial layers on the acellular membrane confirmed the successful construction of organotypic culture model in this experiment.

## 4. Discussions

Based on previous reports, studies comparing the biological seal of soft tissue against zirconia materials under the influence of surface conditioning such as photofunctionalization have not been conducted. Chai et al. [33] have compared the biological seal of titanium with surface modification and surface roughness using a similar technique, where they demonstrated no significant difference in the steady-state flux of four different titanium surfaces, that is, polished, machined, sand-blasted, and TiUnite groups. Given the difference in materials and treatment provided to the surface, direct comparison is not possible. In our study, the concentration expressed as the percentage of HTO that penetrated through the models in 30 min was 1.866 ± 0.217 and 1.522 ± 0.201 for nontreated and UV-treated polished surface, respectively. This value was higher than that reported in the study by Chai et al. [33] during the first hour of HTO permeability time. Given that the surface roughness of titanium samples used in this study was rougher, current findings that the cell especially epithelium was in favor of a smooth surface. The longer junctional epithelial attachment was observed in smooth titanium than the rougher surface, whereas the rougher titanium surface had a long dimension of connective tissue attachment [34,35].

The higher count of HTO in our study could also be attributed to the mode of sterilization of samples before the experimental procedures, that is, ethanol of ascending concentrations was used to remove biological contaminations [36]. Considering that contaminants and chemical debris could significantly change the composition of the surface at the interface level with biological tissues, the cleaning and disinfection techniques adopted before experimental procedures should be carefully selected and performed. In our previous review, we found that alcohol as a mode of sterilization could potentially induce nonbiological contamination, such as hydrocarbon compounds, to the material surfaces [37]. Most studies have shown that surface impurities have been removed following surface photofunctionalization [25,38].

In this study, the concentration of HTO was reduced in the UV-treated group compared with that in the nontreated group. A clean surface had high surface free energy, whereas a contaminated one had low surface free energy. As the surface roughness of the samples in this experiment was standardized, the biological seal formed by the cells was influenced by the surface composition of the material, and surface energy was altered as a result of photofunctionalization. As a semiconductor, zirconia is itself possessed a relatively wide bandgap than that of titanium dioxide, which is around 5.0 eV [23]. The energy photon released by the UV light unit ranges from 3.45–5.0 eV. Therefore, electrons in zirconia can become excited from the valence band to the conduction band by UV treatment provided the photon energy is sufficient. Ultraviolet treatment on zirconia surfaces has shown to induce alteration of physicochemical properties, led to a formation of electropositive surface and enhancement in biologic capability [39]. The divalent cations following UV-treated zirconia surfaces act as direct attractants for cells, and the positively charged zirconia surface can attach directly to negatively charged proteins and cells, leading to improvement in the soft tissue attachment.

Although a large number of studies have confirmed the close association between the stability of the marginal alveolar bone level adjacent to implant and the soft tissue health surrounding the implant abutment [40,41], direct visualization of the region remained technically challenging because of limited research approaches. While the histological en-bloc tissue with in situ abutment is a gold standard in evaluating the nature of peri-implant tissue, the approach is bound to animal and human ethical issues; fixation, and processing technique for ground sections can be daunting because of the contrasting physical properties of hard materials (abutments) and delicate soft tissues. Therefore, this study describes and discusses the validity of an in vitro organotypic oral mucosal tissue used in the investigations to evaluate and quantify the effect of photofunctionalization against materials used for implant abutment. The use of organotypic oral mucosal tissues can provide a comprehensive understanding of oral tissue biology and interaction, including the potential histological assessment of the peri-implant interface compared to simple two-dimensional culture models, which lack the complexity required to draw relevant conclusions. Depending on the purpose of investigations, this approach is an alternative method to animal testing. In most cases, using commercially available tissue construct such as EpiOral™ and EpiGingival™ from MatTek Corp. (Ashland, MA, USA) or SkinEthic™ Reconstructed Human Epidermis (RHE) model (Episkin Laboratories, Lyon city, France) is convenient. These commercial models consist of normal, human-derived keratinocytes, cultured on porous membranes to form a fully differentiated three-dimensional tissue model with or without stratum corneum. Quality controls confirmed the high level of reproducibility and stability of these models over time. Nonetheless, some models used is lacking in fibroblasts and fibroblast-populated matrix, a source of connective tissue cells [42]. The fibroblast component is important not only in promoting the growth and differentiation of keratinocytes into stratified squamous epithelia, but also in ensuring the resemblance of the tissue model to the human peri-implant mucosa and submucosa for the evaluation of the peri-implant interface.

In this study, we have also provided the H&E sections of the remaining tissue following the pull-out of specimen. From these histological sections, we observed that our models lacked stratifications of epithelial cells. Based on the study by Moharamzadeh et al. [43] the thickness of the epithelial layers was influenced by the scaffold used as substrate. Taking into account that the fibroblasts play an important role in epithelial differentiation [33], ensuring that the scaffold is porous enough is important for fibroblast infiltration into the scaffold, thereby enhancing fibroblast–keratinocyte interaction. The acellular human cadaveric dermis used in our study was approximately 0.9 mm thick, a highly porous membrane derived from the human cadaveric skin from which the epidermal layer has been removed. The network of collagen fibers on the basement membrane, collagen scaffold, growth factor receptors, and vascular channels that aid in tissue regeneration is still presented in the membrane. Except for the high cost, using Alloderm^®^ as a collagen substrate in this experiment was a good choice. Moreover, the HGF used in our study were the primary cells to enhance the reproducibility of the oral mucosal model [44,45]. The fibroblasts were used at an early passage number because the extracellular matrix production by fibroblasts decreased as the passage number increased [31]. Hence, the limited stratification of epithelial cells in this experiment might be associated with the duration of the models in the ALI. In all three-dimensional oral mucosal model systems, the keratinocytes exposed to air formed a stratified structure, consisting of several thick cell layers. In most studies, the models were exposed in ALI from 6 days [46,47], 10 days [48], and more than 14 days [49,50]. However, the models developed in our study were increased to ALI for only up to 4 days. This approach could provide an acceptable explanation for the minimal stratification of the epithelium observed. Within the scope of our study, the duration of ALI was considered sufficient [51]. However, in future studies, one could consider a longer period of ALI because the viability of mucosal epithelial cells remained high during this co-culture period, and the total life span of such systems was approximately 5 weeks [52].

As previously mentioned, the cells used in the 3D-PIMM for the permeability test were derived from primary HGK and HGF. Notably, the tissue model systems derived from primary epithelial cells were limited by the donor-to-donor variations in cell growth [51]. In this experiment, a large number of cells particularly primary HGK were obtained from multiple donors. As all tests of each material group and treatment were performed simultaneously, the variation of donors was assumed negligible. Notwithstanding, the stratification of the epithelium affected the permeability, which could be associated with the duration of ALI. This result could be accounted for the slightly higher permeability found in our study compared with that found in previous study [33].

The development of computer-aided design/computer-assisted manufacturing (CAD/CAM) technology has led to the creation of custom-made zirconia abutments, thereby diversifying the prosthetic options of various implant systems to suit the poor angulation of implants and improve their emergence profile [53,54], yet with significant variation in its surface conditions [55,56] which may or may not be suitable for peri-implant mucosal attachment. Moreover, many implant manufacturers produce “copy” design abutments that are claimed to be compatible with well-established manufacturers. These clone abutments will be the subject of biological complications in the future as they may or may not have similar geometry to the original abutments [57,58], thereby compromising the peri-implant tissue attachment. By conditioning these abutments with UV light, nonbiological surface contamination can be reduced or eliminated [25,39]. Based on the result of tissue permeability, we demonstrated that the bioactivity of the zirconia surface is enhanced, and the cell attached is increased in all samples following photofunctionalization. Generally, UV light serves as an energy source for the photocatalytic reaction of both titanium and zirconia where the electron-hole pair is generated and induces the photolysis of hydrocarbon compounds. Both reactions caused a reduction of hydrocarbon compounds and led to superhydrophilicity of the surface. It enhances surface electro-positivity which promote protein adsorption and cellular attachment [15,38].

Other advantages of UV treatment are simplicity of treatment devices and treatment methods, low cost, portability, and diverse applicability for all types of abutment and implant materials. The photo-generated device as shown in Figure 1c is available commercially to treat the implant surfaces at chairside. The exposure time is only 12–15 min [25,38], which is considerably shorter than some devices used by other studies [59,60]. The use of the device is practical clinically, and the UV treatment of implant abutment can be carried out prior to insertion of abutments and final restorations.

## 5. Strengths and Limitations of the Study

This is the first study utilizing the three-dimensional organotypic model to quantify the quality of peri-implant mucosal seal following ultraviolet irradiation of the zirconia surfaces. The use of the three-dimensional oral mucosal model has been utilized to quantify the implant-soft tissue interface endpoints such as in the study of the peri-implant biological seal [33] and soft tissue-implant interface contour [61]. Organotypic cultures best recapitulate the three dimensions of the peri-implant mucosa and can be explored to evaluate many biological endpoints with respect to peri-implant soft tissue interface. It is a reliable, well-defined method and is clinically relevant to study the mechanisms or cellular reaction under normal or influence of different implant surfaces conditions. Thus, the impact of photofunctionalization on the healing of peri-implant–soft tissue against the zirconia material can be ascertained.

Although the use of primary HGK and HGF as a source of cell for three-dimensional organotypic model development simulates clinically relevant information required to study the mechanisms or cellular reaction under normal or influence of different implant surfaces conditions, the cells yield using direct extraction in this study were limited. Therefore, more cells from multiple donors are required for adequate seeding density if multiple experiments were to be carried at the same time. The use of keratinocytes and fibroblasts cell lines will reduce donor’s variation and cells can be consistently passaged in large amount with a high degree of cells reproducibility [43]. Additionally, in our study, the pull tests were performed 14 days after disk insertion, and period of ALI was only up to 4 days, therefore the resultant models have minimum thickness of epithelial layers, thus it may impact the permeability of the model and the specimen–soft tissue interface. Our 3D-PIMM also lacked normal oral cavity environmental conditions, such as the presence of bacterial products that can affect soft-tissue attachment during the inflammatory healing phase [46].

## 6. Conclusions

Using a three-dimensional peri-implant mucosa developed in vitro, we found that the biological seal formed by the soft tissue in a nonphotofunctionalized group was significantly reduced compared with that in the UV-treated group, as demonstrated by the higher concentration of tracing agents penetrating at the tissue interface. Among all the materials tested, zirconia materials exhibited better biological seal than titanium, regardless of surface treatment. Within the limitation of this study, we could conclude that photofunctionalization enhances the soft tissue cell attachment, more so to zirconia materials. Our findings indicated that chairside photofunctionalization is achievable to appropriately modify the surface properties of abutment used in dental implants.

## Figures and Tables

**Table 1:** Surface roughness and composition of materials used in this study.

Materials	Components and Composition by Weight%	Mean Surface Roughness (S_a_ Value ± SD) (nm)	*p*-Value
Commercially pure titanium (CPTi)	TiO^2^	212.05 ± 43.91	0.115 *
Yttria-stabilized zirconia (YSZ)	ZrO_2_/Y_2_O_3_ ≤94%/5.5%	246.48 ± 27.04	
Alumina-toughened zirconia (ATZ)	ZrO_2_/Al_2_O_3_/Y_2_O_3_ 76%/20%/4%	256.65 ± 35.59	

* S_a_ of all the materials was not statistically significant (one way ANOVA at 95% confidence interval). SD = standard deviation; TiO_2_ = titanium oxide; ZrO_2_ = zirconium oxide; Y_2_O_3_ = yttrium oxide; HfO_2_ = hafnium dioxide; Al_2_O_3_ = aluminum oxide.

**Table 2:** Mean percentage of tritiated water (HTO) penetrating through the soft tissue–material interface.

Materials	Mean Percentage of the Amount of Radioactivity that Passed through the Interface (% ± SD)	
	Nontreated Surface (N-Tx)	UV-Treated Surface (UV-Tx)
Commercially pure titanium (CPTi)	1.866 ± 0.217	1.522 ± 0.201
Yttria-stabilized zirconia (YSZ)	1.660 ± 0.017	1.152 ± 0.007
Alumina-toughened zirconia (ATZ)	1.945 ± 0.0137	1.187 ± 0.005
*p* value	0.002 ^#^	

^#^ Permeability of soft tissue at the interface was statistically significant (two-way ANOVA at 95% confidence interval); SD = standard deviation.

## Data Availability

The data presented in this study are available on request from the corresponding authors.
